# Treatment of Foreign Bodies in the Cornea and Their Possible Sequelæ

**Published:** 1907-10-19

**Authors:** 


### OPHTHALMOLOGY.

### TREATMENT OF FOREIGN BODIES IN THE CORNEA AND THEIR POSSIBLE SEQUELÆ.

Foreign bodies, such as small fragments of stone, iron, coal, or dust, etc., in the cornea are of frequent occurrence, and are sometimes followed by serious complications.

The cornea is extremely sensitive, owing to its abundant nerve supply, and the presence of a foreign body gives rise to considerable pain. Large foreign bodies are readily seen, but small ones should be carefully looked for, with oblique illumination of the cornea. Minute foreign bodies—as particles of dust—are readily dislodged from the surface of the cornea by the movements of the lids, and find a resting-place in the upper conjunctival fornix. Here they remain hidden away, and are frequently missed; it is therefore very important to evert the upper lid, and search the upper fornix.

### To Remove a Foreign Body.

The eye is first cocainised, and the conjunctival sac irrigated with boracic acid lotion or normal saline solution. The patient is seated and the surgeon stands behind, steadying patient's head against his chest. The eyelids are kept open by the index and middle fingers of the surgeon's left hand, a gentle pressure being exerted at the same time on the eyeball to control its movements.

Foreign bodies, which are quite superficial, can be readily removed with a clean camel's hair brush. When they are embedded in the corneal substance, they must be gouged out with a special needle or gouge; if these instruments are not available a sewing needle, sterilised in the flame of a spirit lamp, may be used. If a small piece of iron remains embedded in the cornea for some time, the corneal substance in its immediate vicinity becomes impregnated with iron. The brown rust ring thus formed must be carefully scraped away.

When the foreign body has penetrated deeply into the cornea, and is protruding into the anterior chamber, a keratome or broad needle should be passed through the cornea, behind the foreign body. This instrument prevents the foreign body from being pushed into the anterior chamber, and so injuring the anterior capsule of the lens. After the removal of the foreign body the eye should be irrigated with lot. hydrarg. perchlor., 1 in 6,000, and a pad and bandage applied to the eye for a day or two, until the surface epithelium is re-formed.

### After Treatment.

The deeper the foreign body lies, the greater manipulation is required to remove it. This may be followed by an inflammatory reaction, and so gutt. atropine, 1 per cent., should be instilled to lessen the tendency to iritis and diminish the pain, boracic acid lotion used more frequently, and the pad kept on until the ulcer has healed. There is a greater tendency to inflammation if the foreign body has carried with it some septic material. A septic ulcer, with hypopyon iritis, may follow, and careful treatment is then necessary. A similar condition is produced by the infection of the corneal abrasion, by micro-organisms which may be present in the conjunctival or lachrymal sac. Antiseptic lotions are to be used, and if a mucocele is present the lachrymal sac should be syringed out, followed by hot fomentations and gutt. atropine 1 per cent., continued two or three times a day. As long as the ulcer is septic, the hypopyon and iritis continue. Accordingly the floor of the ulcer should be scraped with a small, sharp spoon—the eye is first cocainised, and a drop of fluorescine instilled to mark out the exact area of the ulcer; after a thorough scraping, the floor of the ulcer should be cauterised with pure carbolic acid, or the actual cautery. It may be necessary to repeat this cauterisation and the hot fomentations, and atropine should be continued. A few drops of hydrogen peroxide 8 vols. three times a day, is of value in cleansing the ulcer.

When the ulcer is clean it begins to heal; if it continues foul, and the hypopyon increases, it is necessary to evacuate the pus. This may be done by a Sæmisch's section through the ulcer or by passing a small keratome through the cornea in the lower quadrant. A curette is then introduced into the anterior chamber, the contents of which being thoroughly evacuated. Repeated cauterisation of the ulcer may be done.

Sometimes all treatment fails to arrest the progress of the ulcer, the whole cornea becoming infiltrated and sloughy, ultimately perforating, a panophthalmitis frequently following. As the result may be disastrous, early, careful and proper treatment is most important.

